# Supplementary material for: Are carbon emissions trading and green financial instruments synergistic? -Comprehensive quantitative research based on content analysis
Source: PLoS One. 2024 Mar 7;19(3):e0298601. doi: 10.1371/journal.pone.0298601 (PMC10919601; doi:10.1371/journal.pone.0298601)
Supplement: S1 Data — (ZIP) [file pone.0298601.s001.zip › Data/地级市Z_F工作报告.zip!/║■▒▒/╧σ╤⌠╩╨/└┤╫╘╣1⁄2╓┌║┼í╛┬φ┐╦╩2╛▌═°í┐_2011.docx]

各位代表：

　　现在，我代表市人民政府向大会作政府工作报告，请与《襄阳市国民经济和社会发展第十二个五年规划纲要(草案)》一并审议，并请政协委员和其他列席人员提出意见。

　　一、“十一五”期间的工作回顾

　　过去五年，是我市“九五”以来发展最好最快、人民受益最多的时期。五年来，在省委、省政府和市委的正确领导下，市政府团结和带领全市人民，深入落实科学发展观，围绕提升省域副中心城市综合实力，解放思想，抢抓机遇，奋发图强，全力促进经济社会跨越式发展，全面完成了“十一五”规划确定的主要目标和任务。

　　(一)综合经济实力显著提升

　　经济规模不断壮大。地区生产总值、高新区工业产值、县域工业产值和汽车工业产值相继突破千亿元大关。初步核算，2010年全市生产总值达到1538.3亿元，是2005年的2.59倍，按可比口径计算年均增长14.3%，高于“十五”时期2.3个百分点，占全省的比重由“十五”末的9%提高到9.73%。县域经济规模加速扩张，占全市的比重显著提高。经济效益大幅增长。2010年全市规模以上工业利税总额188.65亿元，是2005年的7.3倍。地方财政总收入141.56亿元，是2005年的2.57倍；一般预算收入51.01亿元，是 2005年的2.77倍，年均增长22.6%，高于“十五”期间11.6个百分点。

　　(二)产业发展水平明显提高

　　经济结构明显改善。三次产业比例由2005年的20.6 ：41.8：37.6调整为15.2：51.9：32.9。

　　新型工业化步伐明显加快。2010年全市规模以上工业增加值681.37亿元，占生产总值的比重由2005年的27.3%提高到44.3%。主导产业发展呈现“一千五百”新格局，汽车工业产值跃上千亿新台阶，食品、纺织、医药化工、建材冶金、电子信息五大产业相继成为百亿产业。我市被国家工信部命名为新型工业化示范基地和军民结合产业示范基地。

　　农业基础地位不断加强。市级以上龙头企业新增108家，达到160家；2010年全市农产品加工业产值662亿元，是2005年的3.59倍。惠农政策全面落实，农田水利等基础设施建设力度不断加大，农业综合生产能力不断提升，粮食生产连续7年增产丰收，畜牧业产值占农业总产值的比重由2005年的33%提高到43%；林业保护和开发力度加大，生态和经济效益不断提高。

　　服务业加速提档升级。商贸、物流市场主体快速发展，万村千乡市场工程和新农村现代物流服务网络工程深入实施，各项促进消费政策全面落实，沃尔玛、鄂武商、万达集团、银泰百货、苏宁电器等一批商业巨头相继入住，有力促进了商贸服务业提档升级。全省家政服务业试点城市建设扎实推进。实施“旅游活市”战略初显成效，一批旅游项目开工建设，旅游收入快速增长。外贸出口规模不断扩大，社会消费品零售总额和外贸出口额年均分别增长18.8%和31%，高于“十五”时期7.7和7.6个百分点。

　　(三)招商引资和项目建设成效显著

　　五年累计新增注册招商项目 2343个，总投资2683.35亿元，东风公司、中航工业集团、航天科技集团、兵器工业集团、兵装工业集团、新兴际华集团、新日电动车公司等一大批知名企业集聚我市；风神襄樊汽车公司天籁轿车扩能项目、骆驼集团新型密闭蓄电池生产线、崔家营航电枢纽等160个亿元以上重大项目建成投产。我市还与3个外国城市结为友好城市，与45个省直厅局开展战略合作。卓有成效的招商引资和项目建设，拉动投资强劲增长，五年累计完成全社会固定资产投资2251.2亿元，是“十五”期间的3.89倍；年均增长39.8%，高于“十五”时期26个百分点。

　　(四)发展方式转变加快

　　科技创新成为经济转型增长的强大动力。以创建国家和省级企业技术中心、工程技术研究中心和创新型企业为重点的创新能力建设成效显著，建立院士专家工作站和服务中心6个，获得省级以上重大科技成果339项，按国家新的认定办法认定高新技术企业74家。新能源汽车、航空航天、生物技术、新能源、新材料等新兴产业呈现良好发展势头，我市成为国家新能源汽车产业示范基地和节能与新能源汽车示范推广试点城市。高新技术产业增加值占规模以上工业增加值的比重由2005年的26.9%提高到2010年的35.3%。我市被评为“全国科技进步示范市”。

　　开发区成为经济集中集约发展的主要平台。争取国家和省批准新建、扩建开发区(工业园区)15个，每个县市区都拥有了产业发展新平台，拉开了工业发展新空间。高新区成为千亿级开发区，国务院批准设立国家级襄阳经济技术开发区，鱼梁洲经济开发区开发建设获得长江水利委员会许可，深圳工业园、航空航天工业园、余家湖工业园等一批特色产业园区发展势头强劲。

　　节能减排成为促进可持续发展的有力措施。节能环保措施不断加强，淘汰落后产能成效显著，单位生产总值能耗下降幅度、化学需氧量和二氧化硫排放量削减幅度均完成“十一五”规划确定目标。环境质量明显改善，汉江市区段水质始终保持二类水平，市区空气质量优良天数比 2005年增加40天，达到316天。我市被评为“全国绿化模范城市”和“全国节水型社会建设示范市”，谷城和老河口、宜城分别成为国家级和省级可持续发展实验区。

　　(五)发展环境不断优化

　　城市承载功能明显增强。城市和城乡交通建设力度加大，市区建成区面积扩大到116平方公里，城市功能、品位和管理水平明显提升，成为省级卫生城市。各县市依托开发区建设新城区，城市面貌呈现新变化。国省干线公路和县乡公路全面升级，经过我市的武(汉)(安)康铁路复线建成通车，汉江航运条件改善，航空运输能力增强，区域综合交通枢纽功能进一步提升。

　　要素支撑能力明显加强。我市成为全省金融信用环境最好的地区之一，国有商业银行不断壮大，5家股份制商业银行相继设立襄阳分行，多种金融业态蓬勃发展，2010年全市金融机构本外币存贷款余额分别达到1294.12亿元和688.25亿元，分别是2005年的2.3倍和2.7倍。资产证券化取得重大突破，回天胶业和台基股份成功登陆创业板，市建投公司成功发行10亿元企业债券。建设用地、煤电油气运等要素供给充足，保障有力。实施“隆中人才支持计划”成效显著，引进创新团队34个，硕士以上高层次人才463人。

　　体制机制明显优化。“阳光新政”深入实施，行政审批制度改革取得突破，通过减程序、减时限、减费用，实现了行政审批“零障碍、低成本、高效率”；以转变职能为核心的市县政府机构改革扎实推进，事业单位分类改革试点工作扎实开展，服务型政府建设步伐加快。国有企业改革、乡镇综合配套改革和集体林权制度改革任务基本完成，农村土地二轮延包依法规范，促进非公有制经济发展的政策措施深入落实，发展活力明显增强。

　　(六)人民生活进一步改善

　　城乡居民收入较快增长。2010年全市城镇居民人均可支配收入13333元，年均增长13.2%。农民人均纯收入6365元，年均增长14.9%，农民收入增长速度连续三年高于城镇居民收入增长速度。

就业和社会保障水平不断提高。全市新增转移农村劳力50万人，城镇新增就业和下岗失业人员再就业68.4万人，城镇登记失业率控制在4.16%以内。社会保障体系建设力度加大，城乡基本医保和城镇低保制度更加完善，农村低保制度、被征地农民和残疾人社会保险制度初步建立，新型农村养老保险试点工作扎实推进，企业离退休人员养老金、城乡低保标准、城镇职工最低工资标准和部分优抚对象待遇大幅度提高。城镇住房公积金制度建设加强，廉租房建设和棚户区改造力度加大，困难户住房租赁补贴制度深入落实，10万低收入居民直接受益。

　　公共服务能力不断增强。义务教育均衡发展力度加大，城镇学校学杂费和农村学校学杂费、书费、住宿费全部免除，进城务工人员子女义务教育权利得到确保，农村“普九”债务全部化解；高中阶段教育基本普及；高等教育发展加快，襄樊学院医学院和襄阳汽车职业技术学院获准设立。医药卫生体制改革稳步推进，城乡卫生服务功能进一步增强，卫生事业发展水平居全省前列，被评为“全国无偿献血先进城市”。省政府下达的计划生育责任目标全面完成。妇女儿童和老年人合法权益得到维护。应急管理、安全生产和消防、食品、药品安全监管全面加强。第六次人口普查取得阶段性成果。

　　新农村建设取得积极成效。累计投资15.67亿元，大力推进新农村建设，13.38万贫困人口的温饱问题和175万人饮水安全问题得到解决，19万个农户用上沼气，所有乡镇和行政村全部通达油(水泥)路和客车，襄南示范区和保康脱贫奔小康试点县建设经验得到省委、省政府充分肯定。

　　(七)民主法制和精神文明建设得到加强

　　民主法制建设深入推进。市政府始终自觉接受市人大及其常委会的法律监督和市政协的民主监督，认真办理人大代表议案、建议和政协建议案、提案；密切联系工会、共青团、妇联、科协等人民团体，广泛听取民主党派、工商联、无党派人士的意见建议，促进了科学决策。加强法制宣传教育，狠抓依法行政目标考核，各级依法行政水平进一步提高。全面推进政务公开，切实加强廉政建设。深入开展社会治安综合治理和信访维稳工作，我市被评为“全国社会治安综合治理优秀市”，信访维稳工作得到中央领导充分肯定。

　　城乡文明程度不断提高。我市被评为“湖北省文明城市”、“全国创建文明城市工作先进城市”和“全国民族团结进步先进市”。国务院批准我市更名为“襄阳市”。文化体育服务体系建设加强，建成各类城乡文化体育设施2525个。文化体育活动丰富多彩，成功举办了第十二届省运会和诸葛亮文化旅游节，我市体育健儿和襄阳籍运动员分别在第十二届、十三届省运会和广州亚运会上取得了突出成绩。“双拥 ”工作进一步加强，我市被评为“全国双拥模范城市”。

　　刚刚过去的2010年，我市经济社会发展取得了显著成绩，地区生产总值按可比口径比上年增长16.2%，规模以上工业增加值、全社会固定资产投资、社会消费品零售总额和地方一般预算收入分别比上年增长26.3%、45.3%、28.2%和37.8%，全社会固定资产投资和规模以上工业增加值均实现两年翻一番；实际利用外资和外贸出口分别比上年增长30%和68.2%；城镇居民人均可支配收入和农民人均纯收入分别比上年增长11.6%和17%；城镇登记失业率控制在4.16%以内。

　　与此同时，援川、援藏、援疆、统计、审计、物价、人防、气象、档案、史志等各项工作都取得了显著成绩。

　　回顾过去五年的政府工作，主要有以下做法和经验值得我们在今后的工作中汲取和坚持：一是立足高远定目标，勇于赶超求发展。几年来，我们坚持高举跨越发展大旗，确定了“四年翻番”的阶段性发展目标，主要经济指标高于全国、全省平均增幅的年度发展目标，以全省争先和三年翻番为重点的县域经济发展目标，提振了信心、鼓舞了士气，凝聚了推进跨越发展的强大合力，全市经济摆脱多年低增长困境，迈入发展的快车道。二是咬定发展不放松，克难奋进往前干。过去五年，面对国际金融危机的冲击和区域经济竞争的巨大压力，我们始终牢牢抓住发展这个第一要务，迎难而上、克难奋进，以开放的视野、改革的举措、市场的办法抓机遇、突困局、干大事、促发展，围绕扩张经济总量，坚定不移抓招商引资、抓项目建设、抓资本市场；围绕打造工业强市，强力实施“三个百亿”和“双千双百”工程，突破性发展新能源汽车和航空航天等新兴产业；围绕发展现代农业，大力实施“一百三千”工程；围绕提升服务业，大力实施“旅游活市”战略，突破性发展商贸物流市场主体；围绕提升城市承载功能，大手笔建设开发区、内外环线等一批重大基础设施，为跨越式发展积蓄了能量。三是着眼民生抓发展，促进发展惠民生。始终把改善民生作为一切工作的出发点和落脚点，在加快经济发展、增强经济实力的同时，不断加大改善民生为主的社会建设投入力度，坚持每年下大力办好一批关系民生的实事，下大力解决人民群众普遍关注的民生问题，努力让全市人民享受更多的发展成果。四是坚持不懈抓环境，凝心聚力干事业。把建设服务型政府作为优化环境的中心环节，深入推进“阳光新政”，提高行政服务能力和水平，有力促进了经济社会发展。把执行力建设作为抓落实、促发展的关键举措，通过实施目标管理，建立激励机制，强化效能监察，促进全市各级凝心聚力干事业，形成了全力以赴抓发展、创先争优比贡献、同心同德促跨越的生动局面。

　　各位代表，过去五年我市经济社会发展取得的显著成绩，是全市人民团结拼搏、开拓创新、奋发进取的结果，凝结着全市人民的智慧和汗水。我代表市政府，向全市人民，向所有纳税人，向各位代表、各位委员，向各民主党派、工商联、人民团体和驻市单位，向驻襄人民解放军、武警官兵，向所有关心支持襄阳发展的同志们、朋友们，表示衷心的感谢，并致以崇高的敬意！

　　回顾过去五年的工作，我们也清醒地看到，困扰我市发展的矛盾和问题仍然较多，主要是：经济规模和实力与中部地区先进城市相比差距仍然较大；经济结构不优，工业主导产业竞争力有待提升，新兴产业刚刚起步；农产品加工业不强，农业基础设施仍然十分薄弱，现代农业还处于初级阶段；现代服务业发展相对滞后，旅游经济刚刚起步；外向型经济、民营经济、县域经济发展水平有待提高；城市功能还不完善，城镇化和新农村建设亟待提速；保障和改善民生的任务还十分艰巨；政府自身建设存在薄弱环节，部分政府部门和公务人员的工作作风、服务质量与人民群众的要求相比还有较大差距。对于这些矛盾和问题，我们将采取积极有效措施，认真加以解决。

　　二、“十二五”经济社会发展指导思想、奋斗目标和战略重点

　　未来五年是我市跨越发展和全面建设小康社会的关键时期。市政府根据市委十一届十二次全会《关于制定襄阳市经济和社会发展第十二个五年规划的建议》精神，制定了《襄阳市国民经济和社会发展规划第十二个五年规划纲要(草案)》，提请大会审议。

　　“十二五”期间，我市处于跨越发展的战略机遇期。工业化、城镇化的快速推进，蕴含着巨大的投资、开发机遇和发展空间；尤其是国家实施促进中部崛起战略、振兴老工业基地以及南水北调工程生态补偿，为我们带来了许多政策机遇；省委、省政府大力推进省域副中心城市建设、鄂西生态文化旅游圈建设和汉江流域综合开发，为我市经济社会发展创造了前所未有的“小气候”；国际和国内发达地区产业转移步伐日益加快，为我们加速要素集聚、扩张经济总量，带来了很多商机；经过“十一五”时期的建设，我市搭建了两个国家级开发区、13个省级开发区等发展平台，培育了一批优势产业和优势企业，加快了交通等基础设施建设步伐，为跨越式发展奠定了坚实的基础。“十二五”时期各种机遇密集叠加，形成了一个大有可为的黄金发展期。我们必须十分珍惜机遇，牢牢抓住机遇，切实用好机遇，奋力推进全市经济社会新跨越，开创富民强市新局面。

　　基于此，《纲要(草案)》提出了我市“十二五”经济社会发展的指导思想和奋斗目标。

　　指导思想是：深入贯彻落实党的十七届五中全会、省委九届九次全会、省委工作会和市委十一届十二次全会精神，以科学发展为主题，以加快转变发展方式为主线，以保障和改善民生为出发点和落脚点，加快经济结构调整优化，推进改革开放和科技创新，努力建成全国重要的汽车及零部件生产基地、高新技术产业基地、新能源汽车产业基地、航空航天产业基地、现代农业基地、区域性综合交通枢纽和物流基地、区域性旅游目的地和鄂西生态文化旅游圈核心基地，进一步增强产业集聚力、科技创新力、环境承载力、文化软实力，把襄阳建设成为综合实力强、辐射带动力大的省域副中心城市，全省重要的核心增长极，鄂豫陕渝毗邻地区的中心城市。

　　奋斗目标是：

　　综合经济实力明显提升。三次产业结构由15.2：51.9：32.9调整为7.6：55：37.4，初步形成具有竞争力的现代产业体系，高新技术产业增加值占规模以上工业增加值的比重达到50%左右。2015年，全市生产总值达到3500亿元，力争4000亿元；地方一般预算收入达到132亿元；五年全社会固定资产投资累计达到8500亿元以上，力争达到9800亿元。

　　城乡现代化建设水平明显提升。构建以中心城市为龙头、大中小城市同步推进、协调发展的城乡一体化发展格局，打造现代化中心城市，形成经济繁荣、功能完善、特色鲜明的城镇体系，城镇化率达到55%以上。农村生产生活条件显著改善，15%的村建成社会主义新农村。

　　民生保障水平明显提升。构建和谐社会走在全国前列，成为全国文明城市、国家卫生城市。居民收入持续增长，城市居民人均可支配收入和农民人均纯收入年均增幅分别达到12.6%和12.8%。城镇登记失业率控制在4.5%以内。覆盖城乡的基本社会保障体系全面建立。逐步解决城镇中低收入家庭的住房困难，城镇家庭入住保障性住房的比例达到15%。物价涨幅控制在全省平均水平以下。城乡居民生活水平不断提高，幸福指数明显提升。

　　可持续发展能力明显提升。人口自然增长率控制在7‰左右；完成国家下达的耕地和基本农田保护任务；非化石能源占一次能源消费比重达到 12%，万元生产总值能耗下降17%；单位生产总值二氧化碳排放降低18%，氮氧化物、化学需氧量、二氧化硫和氨氮排放量控制在省定目标以内，城市生活污水处理率和生活垃圾无害化处置率分别达到95%以上和90%，森林覆盖率达到45%以上，建成国家环保模范城市和国家生态园林城市，让山更绿、水更清、天更蓝。

　　围绕上述指导思想和奋斗目标，“十二五”期间突出抓好八项战略重点：

　　(一)加快构建现代产业体系

　　超常规发展工业。以建成现代工业新城为目标，大力实施“工业强市”战略。做强龙头产业汽车产业，壮大食品、纺织、装备制造、建材冶金四大支柱产业，提升化工、医药、电子电器三大优势产业，突破性发展新能源汽车、航空航天、新一代信息技术、高端装备制造、节能环保、新材料、生物技术等七大战略性新兴产业，力争到2015年，龙头产业汽车工业产值突破2500亿元，食品工业成为千亿产业，新兴产业产值突破千亿元。形成2家过500亿元、10家过100亿元、10家过50亿元、100家过10亿元的骨干企业群；促进国家级襄阳高新区跻身全国20强，国家级襄阳经济技术开发区成为千亿级开发区，县市区开发区工业产值均突破300亿元。

　　加快发展现代农业。以建成全国重要的优质农产品生产加工基地为目标，加快推进农业生产大市向现代农业强市跨越。突破性发展农产品加工业，培植和引进一批产销过10亿元、50亿元乃至过百亿元的龙头企业，创建一批百亿加工园区和百亿加工县市。按照“优质化、规模化、标准化、专业化”的要求，重点建设小麦、水稻、油料、蔬菜、茶叶、干鲜果、烟叶、棉花、畜禽和种子(种畜)等十大农产品生产板块；巩固提高国家商品粮基地建设水平，建成全省乃至全国知名的规模化畜禽养殖基地、特色淡水渔业基地和特色林产品生产加工基地。努力实现百亿斤粮食产能、千万头牲畜出栏量和千亿元农产品加工产值的发展目标，为建成千亿元林产业奠定坚实基础。

　　突破性发展现代服务业。一是突破性发展文化旅游业。大力实施“旅游活市”战略，强化襄阳市区旅游龙头地位，重点建设古隆中、习家池、襄阳古城、岘山森林公园、鹿门山以及南漳古山寨、保康九路寨和谷城大薤山(南河)八大景区，规划建设鄂西北旅游服务中心，努力把襄阳建成区域性旅游目的地和鄂西生态文化旅游圈核心基地。二是加快发展现代物流和商贸服务业。依托综合交通运输体系，促进现代物流和商贸服务业突破性发展，逐步把我市建成全国物流信息交换中心、中部地区物流节点城市和鄂豫陕渝毗邻地区商贸物流中心。三是大力发展新兴服务业。以建设区域性信息服务中心节点城市为目标，推进“数字襄阳”和“智能城市”建设，加快发展信息服务业。积极发展创意产业、应用软件开发和服务外包产业、通用航空服务业、社区服务业，以及工程咨询、科技服务、市场中介、法律、会计、评估等中介服务业。四是规范发展房地产业。认真执行国家房地产政策，重点建设公租房为主的保障性住房、经济适用房和普通商品房，努力缓解中低收入家庭住房困难，促进房地产市场健康有序发展。

　　(二)加强基础设施和基础产业建设

　　抢抓国家加大交通建设力度的机遇，加快建设高速铁路、高速公路、县乡公路、汉江航运和机场口岸为重点的现代综合交通运输体系，支持建成郑(州)渝(重庆)高速铁路，争取建设西(安)武(汉)高速铁路，提升我市区域交通枢纽地位。抢抓我国未来十年大兴水利的重大机遇期，加快建设农田水利、防洪设施和城乡供排水设施，构建现代水利体系。抢抓国家加强能源建设的机遇，加快发展能源产业，大力发展火电、水电、生物质能和太阳能；加快改造城乡电网，建设坚强智能电网；加快推进煤炭供应基地、储运中心和油气设施建设，增强能源保障能力。

　　(三)加速城镇化和城乡一体化进程

　　加快构建充满活力的城镇体系。着眼于建设区域性中心城市，依托山、水、城、洲、文化资源禀赋和内外环线，大手笔、高品位规划建设新襄阳，构建“一线穿珠、二线环绕，一江(汉江)两河(唐白河、小清河)两山(岘山、鹿门山)”和“一洲(鱼梁洲)四城(襄城、樊城、襄州、东津)”的空间布局，依托近郊城镇规划建设卫星城市，推进南漳、宜城与市区一体化，努力把襄阳建成经济繁荣、功能完善、环境优美、宜居宜业宜旅的现代化大城市。支持枣阳建成50万人以上的中等城市，支持谷城、老河口组团发展为50万人以上的中等城市，支持保康建成生态旅游、现代农业和农产品加工基地，支持 12个全省城乡一体化试点镇建成5万人左右的小城市。创新体制机制，完善政策措施，提升城镇功能和产业集聚度，引导农村人口向城镇有序转移。

　　加快建设社会主义新农村。大力推进中心村建设，建成一批农村新型社区，逐步实现农村基础设施城镇化、生活服务社区化、生活方式市民化、基本公共服务均等化。突出抓好襄南、鄂北和石花示范区，汉江、铁路和国省道沿线试点村，以及“口子镇”、乡镇政府所在集镇为重点的新农村建设，带动全市新农村建设上水平。

　　(四)促进区域协调发展

　　大力支持县域经济跨越式发展。每个县市区形成1-2个竞争力强的主导产业，建成一批税收过千万元、过亿元的骨干企业，力争1-2个县市区进入“中部百强县市”行列，3-4个县市区进入全省县域经济发展20强。完成农村最低生活保障制度和扶贫开发政策衔接，加大对革命老区、少数民族地区和贫困山区的扶持力度，帮助36.9万农村贫困居民摆脱贫困；扎实开展援藏、援疆工作。按照国家和省主体功能区规划要求，优化国土空间布局。

　　(五)建设资源节约型和环境友好型社会

　　严格落实节能减排目标责任制，大力实施节能减排工程，确保节能减排目标全面实现。实行最严格的土地、水资源管理制度，促进集约节约用地、用水。大力发展循环经济和低碳经济，抓好国家再生资源试点城市建设，打造节能产业园区和再生资源基地；倡导绿色消费，建设低碳城市。加强生态环境保护与建设，严格实施项目建设环评制度，汉江干流水质稳定在省控功能区划标准以上；有效控制温室气体排放，改善大气环境质量；全面开展农村环境综合整治，防治农业面源污染；大力发展碳汇林，积极创建国家和省级自然保护区，加强天然林区、重要水源涵养区、石漠化地区的生态保护和治理，促进生态修复。

　　(六)促进经济社会协调发展

　　加快推进创新型襄阳建设。大力普及科学技术，着力营造创新氛围。建立多元化投融资体系，确保科技创新投入持续增长。完善企业为主体、市场为导向、产学研相结合的技术创新体系，力争在促进新兴产业领域科技创新、培育创新型企业以及争创国家和省级技术研发机构上实现大突破，把国家级高新区、国家级经济技术开发区建成全省科技创新和新兴产业发展示范区。

　　加快推进教育改革和发展。坚持教育优先发展的方针。基本普及学前教育。初步实现县域义务教育均衡发展。全面普及高中阶段教育。加快发展特殊教育。积极发展民办教育。大力发展高等教育和职业技术教育，努力把襄樊学院建成具有特色优势的综合性大学，把襄樊职业技术学院建成一流的国家示范职业技术学院，加快建设襄阳汽车职业技术学院和襄樊学院理工学院，积极创建国家和省级中等职业示范学校。

　　加快实施“人才强市”战略。创新人才培养、选拔、激励和评价机制，形成有利于优秀人才脱颖而出的良好环境。着力引进、培养、聚集一大批精通国际规则、具有世界眼光的高层次领军人才、创新人才和团队，为全市经济社会发展提供有力的人才和智力支撑。

　　加快发展文化事业。适应群众精神文化需求新变化、新要求，弘扬主旋律，提倡多样化，大力支持创作先进文化精品。深入推进全国文明城市和全省文化事业示范基地创建工作，加强文化体育设施建设，繁荣城乡公益性文化体育事业，丰富社会文化生活，全面提高城乡居民文明素质。大力发展文化产业，加快建设具有地方特色的文化产业基地和产业集群。

　　(七)深入推进改革开放

　　深入推进各项改革。坚持和完善基本经济制度，鼓励全民创业，突破性发展非公有制经济。深化国有企业股份制改造，完善国有资产管理制度，确保国有资产保值增值。稳定和完善农村基本经营制度，推行土地承包经营权流转，完善集体林权制度，抓好国有农场体制改革，完善农村公益性服务“以钱养事”新机制，增强农业和农村经济发展活力。加快推进投资融资体制改革，着眼于建设区域性金融中心，大力发展银行、证券、保险等各类金融组织，完善地方投融资平台，形成多元化、高效率的融资服务体系，力争在推进企业上市和债券融资上实现大突破。按照中央和省统一部署，推进水、电、气、热等资源性价格和环境收费改革，促进可持续发展。加快推进政府自身改革，以“阳光新政”工程化为重点，加快转变政府职能，着力建设服务型政府。以加强目标管理为重点，深化政府管理创新，着力建设效能政府；健全公共财政体制，强化财政收支管理；加强廉政建设，树立廉洁从政、勤政为民的政府形象。

　　积极扩大对外开放。加大对外交往交流力度。加快发展外向型经济。坚定不移地强化招商引资，推进项目建设，科学谋划实施一批关系全局和长远发展的重点项目，确保“十二五”规划项目库入库项目总投资达到2万亿元以上；抢抓国内外产业转移和央企扩张机遇，力争把我市建成全国知名的承接产业转移基地、中央企业战略布局的重点区域。大力发展国际贸易，加快建设优势产品出口基地，着力培育出口品牌和骨干企业；加强保税库和监管库建设，积极申报建设保税物流中心；完善铁路、公路口岸功能，争取设立航空和水运口岸，优化开放环境。实施“走出去”战略，鼓励企业利用国外资源和国际市场加快发展。

　　(八)着力保障和改善民生

　　坚持把保障和改善民生作为公共财政优先方向。采取更加积极的就业政策和措施，促进社会充分就业，构建和谐劳动关系。建立健全覆盖城乡的社会保障体系，全面推进城镇企业职工养老保险省级统筹和医疗、失业、工伤、生育保险市级统筹，完善城乡居民医保制度，全面推行城乡最低生活保障制度和新型农村养老保险制度，健全城乡一体化社会救助体系。合理调整收入分配关系，认真落实国家收入分配政策，动态调整最低工资标准，帮助低收入家庭开辟增收渠道，创造条件增加居民财产性收入。以实现人人享有基本医疗卫生服务为目标，推进医药卫生体制改革，建立健全覆盖城乡的公共卫生服务、医疗服务、医疗保障和药品供应保障体系，促进基本公共卫生服务逐步均等化。加强人口和计划生育工作，稳定低生育水平，提高出生人口质量，促进出生人口性别比趋于正常。大力发展老龄事业、妇女儿童事业和残疾人事业。

　　推进社会管理创新。建立党委领导、政府负责、社会协同、公众参与的社会管理格局，创新社会管理体制。加大城市社区和农村新型社区建设力度，培育发展各类社会组织，提高社区自治水平。完善社会矛盾纠纷调处机制，切实加强信访维稳工作和社会治安综合治理，着力建设“平安襄阳”。广泛开展法制宣传教育，坚持依法行政，促进司法公正，打造法治政府，建设“法治襄阳”。加强防灾、抗灾、减灾和救灾体系建设。建立健全公共安全事件预警应急机制，提高紧急应对能力。认真落实企业安全生产主体责任，加强安全生产监察监管能力建设，确保安全生产主要指标控制在省政府下达目标以内。狠抓消防、食品、药品及医疗安全监管，保护人民群众生命和财产安全。加大人防工程和国防后备力量建设力度，加强“双拥”工作，巩固军政、军民团结好局面。依法管理民族和宗教事务，促进各民族团结和谐。

　　三、2011年政府工作目标和任务

　　2011年是实施“十二五”规划的起步年，市政府将紧紧依靠市委的坚强领导和市人大、市政协的监督支持，团结和带领全市人民，高举跨越发展旗帜，强化跨越发展举措，奋力实现“十二五”良好开局。

　　今年全市经济社会发展的预期目标是：地区生产总值增长14.5%以上，规模以上工业增加值增长20%以上，全社会固定资产投资增长30%以上，社会消费品零售总额增长20%以上，实际利用外资和出口额分别增长15%和20%，地方一般预算收入增长21%以上，城镇居民人均可支配收入和农民人均纯收入分别增长11%和10%以上；城镇登记失业率控制在4.5%以内。全面完成省政府下达的计划生育、节能减排等约束性目标。

　　为实现上述目标，我们将重点做好八个方面的工作：

　　(一)大力推进招商引资和项目建设

　　切实把转变发展方式的措施落实到支撑科学发展的项目上来，全社会固定资产投资突破千亿元。

　　加快建设重点项目。重点抓好166个亿元以上项目建设，突出抓好43个投资过10亿元的重大项目，完成亿元项目投资400亿元以上，新投产项目60个以上。争取郑(州)渝(重庆)高铁襄阳段开工建设，支持开展西(安)武(汉)高铁襄阳段规划研究；争取新集电站开工建设，大唐热电项目获得国家批准。强化项目建设推进措施，严格考核各地用地量、中长期贷款增量、用电报装量和接电量以及与投资相关税收增量，加大项目建设推进力度。

　　抢抓国家投资项目。抓紧与中央积极的财政政策对接，加大中央和省投资项目争取力度，力争在“三农”、水利、教育、卫生、城乡社保、城乡居民医保、保障性住房、城市交通和新兴产业等方面，有一大批项目列入国家和省投资计划。确保我市在全省争取中央和省项目、资金的份额不减，力争稳中有升。

　　抢抓重大招商项目。进一步完善招商工作机制，狠抓驻点招商、专业招商、以商招商，鼓励全民招商。加强与有重组扩张计划的央企对接，与实施产业整体转移的发达地区对接，与寻找项目的发达地区民间资本对接，与寻求扩张的国内外大公司对接，全力引进大企业，建设大项目，力争每个县市引进1个投资过10亿元、2个投资过5亿元的工业项目，全市招商项目投资占全社会固定资产投资的比重达到35%。全面落实相关政策，鼓励全民创业，促进民间投资，加快发展非公有制经济，使之成为项目兴市的重要力量。

　　保障项目建设要素供给。积极争取金融机构支持符合国家产业政策的产业发展和项目建设。争取一批新的股份制银行和村镇银行正式运行，积极引进创投公司、担保公司、信托公司、小额贷款公司，运用多种金融工具，扩大金融市场规模。力争金融机构贷款余额突破900亿元。争取在推进企业上市和进军“新三板”市场上取得突破，抓好中小企业集合债和市城投债二期发行工作。做强城投和旅投公司，争取交通投资公司正式运作，优化投融资平台。强化集约节约用地，促进工业项目建设多层厂房；整治违法用地，抓好土地整理，盘活存量土地，加大建设用地报批力度，保障重点项目用地需求。

　　(二)强力实施“工业强市”战略

　　大力推进“双千双百”工程，加快建设千亿产业和千亿开发区，加快打造百亿企业和百亿园区。

　　加速推进产业扩张。力争完成工业项目投资530亿元以上，强力推进结构调整，扩张产业规模。全面落实与东风公司的战略合作协议，全力支持东风公司加快实施高档乘用车和发动机扩能等一批总投资120多亿元的项目；依托东风整车产能优势，突破性发展汽车零部件产业。以建设国家军民结合产业基地为契机，积极争取中航工业集团、航天科技集团、兵器工业集团、兵装工业集团、新兴际华集团加快实施在我市总投资200多亿元的项目。加快建设龙蟒集团磷酸盐、美亚达公司彩涂铝板、襄大农牧公司冷鲜食品加工等72个投资过亿元的支柱产业项目，壮大汽车、食品、纺织、建材冶金、化工、医药、电子电器、装备制造和能源电力等支柱产业集群。力争九大产业产值突破2800亿元。加快实施新兴产业行动计划，突出抓好东风新能源客车、新日电动车、珠江数控机床、高迪数码相机、中日龙工业机器人等33个投资过亿元的新兴产业项目建设，促进新能源汽车、航空航天、高端装备制造、新一代信息技术、节能环保、新材料等新兴产业突破性发展；加快建设国家新能源汽车产业示范基地和国家动力蓄电池检验检测中心，抓好国家节能与新能源汽车推广示范城市试点工作，带动新能源汽车整车及电池、电机、电控等关键部件研发生产，培育新的经济增长点。

　　加速推进企业成长。深入实施企业成长工程和全民创业工程，促进中小企业加快成长；实施大企业培育计划，支持骨干企业做大做强。力争今年销售收入2000万元以上的规模企业新增170户，达到1100户；产销过亿元和10亿元的企业分别新增40户和8户，促进骆驼集团、奥星粮油、中航精机3家企业产销突破50亿元。实施“质量兴市”战略，力争新增一批省级以上精品名牌产品和驰名商标。加强工业企业公共信息化服务平台建设，加快推进工业化与信息化融合。强化煤电油气运等要素协调，加快建设储煤中心和稳定的煤源基地，加强城乡电网建设，千方百计满足工业运行需求。

　　加速发展开发区。抓紧做好高新区扩区规划编制和报批工作，拓展发展空间，力争在引进世界500强、中国100强企业上取得新突破。抓紧编制国家级经济技术开发区发展规划，加快提升深圳工业园产业集聚功能，力争新增注册投资过亿元的工业项目20个以上，新投产项目30个以上。加快建设余家湖工业园和航空航天工业园基础设施。大力发展新兴重工工业园、江山重工产业园、华中制药产业园、三环襄轴工业园、同济堂产业园、奥瑞那国际光电子产业园等特色产业园区。支持县市开发区加速向百亿园区迈进。

　　(三)加快发展现代农业

　　深入实施“一百三千”工程，加快推进百亿斤粮食产能、千万头家畜养殖能力、千亿元农产品加工业和千亿元林产业建设。

　　加快推进农产品加工业突破性发展。支持市级以上龙头企业以品牌为标志、以产权为核心、以深加工为目标加速扩张，引进一批知名企业开工建设，促进相关产业加速整合，力争创建 1-2个百亿加工县市、2-3个省级林业科技产业园区，着力打造全国知名的农产品品牌。全市农产品加工产值达到750亿元。

　　加快推进农业板块基地建设。大力实施百亿斤粮食产能建设工程，启动“一江(汉江)一岗(鄂北岗地)”综合开发，粮食总产稳定在90亿斤以上。扩大蔬菜复播和油料种植面积，积极发展烟叶、棉花、水果、茶叶、食用菌等特色产业，加快建设木本油料林、生物质能源林、速生丰产林、高效经济林等林业板块基地。突破性发展畜禽养殖业，创建一批全省重点养殖园区，建成4个畜牧大县、5个生猪调出大县、10个畜牧养殖大镇。积极探索农村土地经营权流转，促进农业规模化经营。大力实施农民专业合作社“一村一社”发展计划和示范社建设工程，提高农业和农民组织化程度。

　　加快推进农业科技创新。大力推广使用优质良种，集成应用先进生产技术，抓好全国农机化示范区项目建设，提高农业生产科技含量。以创建全国农业标准化示范市为目标，抓好43万亩标准化生产基地建设，加强农产品质量安全检测，新增有机、绿色、无公害农产品认证标识40个。抓住国家支持生物育种业发展的契机，力争在发展粮油作物育种、花卉苗木及畜禽良种繁育产业上取得突破。

　　加快推进水利等基础设施建设。加快推进小型水库除险加固、大型灌区整治、“一江两河”堤防标准化整治和南水北调补偿工程，启动汉江贾家洲洲滩整治项目。加快实施鄂北水源工程，抓好“小农水重点县”建设，扩大高产农田规模。认真做好水利普查工作。

　　(四)突破性发展服务业

　　突破性发展文化旅游业。加快建设八大景区旅游项目，启动建设鱼梁洲经济开发区基础设施和旅游项目，扎实抓好汉十线襄阳段生态景观工程和城市外环线绿化景观工程；着力打造释道安文化旅游品牌；力争创建国家4A景区3个以上。系统策划营销襄阳旅游，努力提升襄阳旅游的美誉度和影响力。

　　突破性发展现代服务业。加快推进传统商业网点升级改造，高标准规划建设一批城市商业综合体，支持樊城区创建国家级现代服务业示范区。扶持发展城市社区连锁便利店和家政服务业。深入落实中央扩大消费政策措施，加快推进万村千乡市场工程和新农村现代流通网络工程，开展大型农超对接活动，促进城乡消费升级。加快发展现代物流业，着力培植骨干物流企业，规划建设牛首、襄北等物流园区。充分发挥保税库和监管库的作用，推进口岸“大通关”建设，积极申报设立航空口岸，扩大对外开放。积极发展服务外包、电子商务、信息服务等现代服务业。

　　(五)加速推进城镇化

　　加快发展中心城市。着眼于建设鄂渝陕豫毗邻地区中心城市，高水平规划和建设中心城区。突出抓好东津现代新城、鱼梁洲生态花园城、庞公滨江新城、襄南大学城、经济技术开发区和大型城市综合体的规划或建设，逐步形成具有襄阳特色的城市型态和地标性建筑群。加快改造“城中村”和城市棚户区。以打造城市景观为重点，推进城市森林生态工程建设、汉江岸线开发以及南湖宾馆至襄阳机场和凤凰温泉景观路综合整治。以解决交通拥堵为重点，加快推进内、外环线建设和市区干道改造，构建城市骨干交通体系。以提升城市功能为重点，加强防洪排涝、污水收集处理、供水供气、环卫等基础设施建设。以创建全国文明城市、国家环保模范城市、国家卫生城市、国家生态园林城市和“楚天杯”为抓手，加大城市管理和环境综合整治力度，依法制止违法建设，启动城市数字化管理，提高城市管理水平。大力推进通达县市的高速公路和国省干线建设，构建市域快速交通圈。

　　加快建设新型城镇体系。着眼于打造中等城市，高起点规划建设县城。重点建设以开发区为核心的新城区，完善城市功能，优化人居环境，增强要素吸纳力，加快推进产业集聚，壮大县域经济中心。以12个全省城乡一体化试点城镇为重点，加快培育各具特色的小城市。推进城镇资源和资产经营，运用市场手段筹集建设资金。

　　加快建设社会主义新农村。坚持“片线面结合、市县镇联动”的思路，整体推进全市新农村建设。以创建全省“宜居村庄”为重点，加快建设中心村，实施“一建三改”工程，加大水利、道路、电力、通信、垃圾和污水收集处理等基础设施建设力度，大力发展公共事业。抓好移民新村建设。办好鄂北城乡一体化试点，探索城乡一体化发展的有效途径。

　　加快建设资源节约型和环境友好型社会。严格落实节能减排目标责任制。加大创建国家环保模范城市工作力度。加强以汉江及其主要支流为重点的水污染防治，确保我市汉江水质稳定在现有水平，保持在国家规定标准以上。严格控制新建高耗能、高污染项目，加快淘汰落后产能。完成市区张湾截污干管、隆中污水治理项目和太平店污水处理厂建设任务，建成市区垃圾焚烧发电项目。积极做好应对气候变化工作。推进农村环境连片整治，支持谷城、保康创建国家级生态示范县。强化节能降耗和节地、节水措施，提高能源资源利用效率。抓好国家再生资源回收利用体系建设试点工作，推进10个国家和省级循环经济试点建设，加快发展循环经济。

　　(六)深入实施“科教兴市”和“人才强市”战略

　　大力推进科技创新。加快培育创新型企业，支持国家和省级创新型试点企业加快发展。创新产学研合作方式，鼓励企业与高校联合建设院士工作站、博士后工作站、工程技术研究中心，创办科研生产联合体，力争全年开展产学研合作项目100个。普及科学技术，鼓励科技创新，力争在知识产权创造、运用、保护和管理上取得新突破，申请专利2600件。

　　大力发展教育事业。坚持优先发展教育事业。科学编制中长期教育改革和发展规划。积极办好学前教育。推动义务教育均衡发展，提高农村中小学生均公用经费标准，确保留守儿童、流动人员子女、残疾儿童、城乡贫困家庭学生公平接受义务教育。推进普通高中优质化。大力发展职业教育，完善现代职业教育体系。壮大高等教育规模，支持襄樊学院办好医学院，争取组建农学院；支持襄樊职业技术学院建设国家示范职业技术学院，加快建设襄阳汽车职业技术学院和襄樊学院理工学院，争取襄阳技师学院建设取得突破。

　　大力实施人才强市战略。创新机制，加大投入，优化人才发展环境。深入实施“隆中人才支持计划”，紧紧围绕产业发展需求，实施重大人才工程，力争面向海内外引进一批紧缺急需的高端人才和创新创业团队。

　　(七)着力加强民生和社会建设

　　切实保障和改善民生。一是千方百计扩大就业。全面落实国家促进就业政策，积极创建省级创业型城市，推进创业带动就业，大力促进高校毕业生就业，积极帮助困难人员稳定就业，促进社会充分就业，城镇登记失业率控制在4.5%以内。二是着力提高社会保障水平。争取扩大新型农村养老保险试点覆盖面，提高企业退休人员养老金标准；启动城镇医疗、工伤、生育保险市级统筹工作，提高城乡基本医疗保险参保率和保障水平，实现参保人员住院费用即时结算。加大城镇医疗救助力度。免除困难群众基本殡葬服务费用。加快建设城市廉租房和公共租赁房，增加普通商品住房供给，发挥住房公积金制度的保障作用，提高住房保障水平。进一步加大扶贫助残力度。大力发展养老事业，切实关心农村空巢老人和留守儿童。

　　三是加强市场保障和价格稳定工作。确保生活必需品市场供应充裕，稳定市场价格；建立低收入群体价格动态补贴机制；开展重点行业收费专项检查，及时依法纠正不规范、不正当价格行为。确保居民消费价格指数涨幅控制在全省平均水平以内。

　　加快发展社会事业。一是促进文化大发展大繁荣。加大文化体育设施建设力度，鼓励创作文艺精品，实施送戏、送书、送文化下乡等文化惠民工程，开展丰富多彩的群众文化和全民健身活动；整治文化市场，重拳扫黄打非，净化文化环境。突破性发展文化产业，加快推进米芾国际文化创意园、襄阳影视基地、中国东汉文化城等重大文化产业项目建设；鼓励社会力量创办文化企业，推进经营性文化事业单位转企改制，增强文化产业发展活力。二是加快发展卫生事业。深化医药卫生体制改革，扩大国家基本药物制度试点范围，积极稳妥推进公立医院改革；加强以乡、村两级医疗卫生机构为重点的基层医疗卫生服务体系建设；加大重大传染病防治力度，积极发展无偿献血、妇幼保健和疾控、急救等公共卫生事业。三是加强人口和计划生育工作。稳定现行生育政策，促进出生性别比趋于正常，不断提高人口素质。

　　加强和创新社会管理。完善城市社区管理体系，加强农村新型社区建设，抓好第八届村民委员会和第四届居民委员会换届选举工作，建成100个村务公开和民主管理示范村(居)委会，提高社区自治水平。健全群众权益维护机制，把各种不稳定因素化解在基层和萌芽状态。强化社会治安综合治理，严厉打击各类刑事犯罪，努力提高人民群众对社会治安的满意度。支持国防建设，加强“双拥”工作。进一步提高应急管理能力，加强各种自然灾害防灾减灾救灾工作。全面落实企业安全生产主体责任，深化隐患排查治理和专项整治，打击非法生产行为，强化安全基层基础，提高安全监管、安全保障和救援能力；依法加强消防、食品、药品和医疗安全监管，保障人民群众健康和生命安全。

　　在做好以上各项工作的同时，市政府将着力办好关系民生的十件实事。

　　1、积极扩大就业，新增城镇就业岗位7.5万个，开发公益性岗位3000个；培训城乡劳动力8万人，新增转移农村劳力4万人。

　　2、新建和改扩建市区20个社区服务中心，建设市行政服务中心，提高便民服务水平。

　　3、提高社区工作者、环卫工人等低收入工作者工资待遇；扩大农村低保覆盖面，提高城乡低保和农村五保对象补助标准和机构供养孤儿最低养育费用；将“五七工”、“家属工”和困难集体企业职工纳入养老保险。

　　4、基本实现对所有贫困学生提供资助；70%以上的乡镇各办1所公立幼儿园。

　　5、实现0-14岁儿童扩大国家免疫规划全覆盖；开展贫困残疾人关爱行动，实施0-6岁残疾儿童抢救性康复治疗工程。

　　6、筹集市区公共租赁住房和廉租房房源4000套，对人均住房面积13平方米以下的困难家庭发放租赁补贴，缓解住房困难。

　　7、完成惠及36.1万户的农村电网改造工程，完成48座病险水库除险加固工程，兴建1000个以上村级综合服务社和农家店；实施新一轮整村推进扶贫计划，完成850个贫困户扶贫搬迁任务，解决3万人贫困人口的温饱问题。

　　8、为城乡困难群众办理法律援助案件2500件以上。

　　9、新建城市社区流动图书站30个、农家书屋400个、城乡体育健身设施100个。

　　10、新建、改建樊宛路、邓曼路等20条市区道路和22公里配套排水管网，新建紫贞公园、民城路绿化带等20万平方米城市绿地，提升城市宜居水平。

　　(八)切实加强政府自身建设

　　深入推进解放思想。紧紧围绕跨越式发展目标，推动新一轮思想大解放。进一步强化竞争意识，勇于在全省、全国找标杆，以高远的目标引领发展大跨越；强化开放意识，拓宽发展视野，以大开放推进发展大跨越；强化创新意识，发扬敢闯敢试、敢于负责的精神，以改革的举措、市场的办法促进发展大跨越。

　　推进“阳光新政”。大力推进“阳光新政”工程化，建立行政审批、市场监管、公共服务、社会监督“四位一体”的运行平台，逐步实现行政服务标准化和信息化；大力推行电子政务，加强电子监察，深化政务公开，规范行政行为。以“治庸治懒、提能增效、狠抓落实”为重点，加强机关作风和效能建设。建立重大决策部署执行情况定期检查和专项督查制度，完善政府目标管理机制，认真落实行政问责制，促进形成雷厉风行、不折不扣抓落实的好风气。

　　加强民主法制建设。深化统计制度改革，加强经济社会运行情况分析和研究，认真执行重大事项专家论证和公开听证制度，广泛听取各民主党派、工商联、无党派人士以及社会各界对政府工作的意见建议，提高科学决策水平。认真执行市人民代表大会及其常务委员会各项决议，主动加强与人民政协的协商，自觉接受市人大的法律监督和市政协的民主监督，认真办理人大代表议案、建议和政协建议案、提案，促进政府工作水平不断提高。推进依法行政，促进司法公正，着力建设“法治襄阳”。

　　坚持从严治政。认真执行党风廉政建设责任制，教育和引导政府公务人员自觉做到廉洁从政。加强对招投标、政府采购、财政和社保资金使用、惠农资金发放、行政事业性收费以及行政处罚的过程监管，认真开展公务用车问题专项治理，着力解决关键领域、关键岗位的腐败问题，树立廉洁政府的好形象。

　　各位代表，新的五年，新的征程。我们肩负着建设更加繁荣富强新襄阳的历史任务，肩负着全市人民对幸福美好新生活的殷切期待。让我们在市委的正确领导下，深入贯彻落实科学发展观，解放思想，振奋精神，开拓进取，扎实工作，为实现襄阳经济社会跨越式发展而努力奋斗！
